# JNK Pathway-Associated Phosphatase/DUSP22 Suppresses CD4^+^ T-Cell Activation and Th1/Th17-Cell Differentiation and Negatively Correlates with Clinical Activity in Inflammatory Bowel Disease

**DOI:** 10.3389/fimmu.2017.00781

**Published:** 2017-07-04

**Authors:** Rui Zhou, Ying Chang, Jing Liu, Min Chen, Hongling Wang, Meifang Huang, Shi Liu, Xiaobing Wang, Qiu Zhao

**Affiliations:** ^1^Department of Gastroenterology, Zhongnan Hospital of Wuhan University, Wuhan, China; ^2^The Hubei Clinical Center and Key Laboratory of Intestinal and Colorectal Diseases, Wuhan, China

**Keywords:** JNK pathway-associated phosphatase, inflammatory bowel disease, CD4^+^ T cell, activation, proliferation

## Abstract

This study aimed to investigate the role of JNK pathway-associated phosphatase (JKAP) in inflammatory bowel disease (IBD). JKAP expression was analyzed in the intestinal mucosa of 81 IBD patients and 25 healthy controls (HCs) by qPCR and immunoblotting. The correlations of JKAP with clinical activity and inflammatory cytokines were performed. JKAP expression before and after infliximab treatment was also measured. CD4^+^ T cells were isolated from peripheral blood in active IBD patient and HCs and transduced with lentivirus-encoding JKAP (LV-JKAP), anti-JKAP (LV-anti-JKAP), or empty vector (LV-scramble), and JKAP functions on IBD CD4^+^ T cells were subsequently investigated. JKAP expression was decreased in inflamed mucosa of active IBD patients and was negatively correlated with disease activity [Crohn’s disease activity index (CDAI), Mayo index, C-reactive protein, and erythrocyte sedimentation rate], interleukin-17, and tumor necrosis factor (TNF)-α levels. Anti-TNF-α treatment up-regulated JKAP expression in CD patients, and baseline JKAP expression was elevated in response patients than in failure patients. Transduction of LV-JKAP into CD4^+^ T cells inhibited the percentages of CD25^+^ and CD69^+^ cells and proliferation. Moreover, inhibition of JKAP promotes Th1/Th17 cell differentiation. Our data indicated that the decreased expression of JKAP in intestinal mucosa contributed to the pathogenesis of IBD, through facilitating CD4^+^ T-cell activation, proliferation, and Th1/Th17-cell differentiation.

## Introduction

Inflammatory bowel disease (IBD), mainly including Crohn’s disease (CD) and ulcerative colitis (UC), is a common gastrointestinal disorder worldwide (1, 2). Although the exact etiology of IBD is still unknown, IBD has been considered to result from the combination effect of genetics, immune responses, and environment factors (2–4). Accumulating evidence has demonstrated that CD4^+^ T cells are critical for the development of chronic intestinal inflammation, and CD4^+^ T-cell-related cytokines, such as tumor necrosis factor (TNF), interferon (IFN)-γ, interleukin (IL)-12, and IL-17A, are up-regulated in the inflamed mucosa of patients with IBD (5–7).

Dual-specificity phosphatase (DUSPs), which could dephosphorylate both tyrosine and serine/threonine residues, are involved in several biological behaviors (8). JNK pathway-associated phosphatase (JKAP), which is also known as DUSP22 belonging to the low molecular weight atypical DUSPs family, is extensively expressed in various types of mammalian cells such as T cells, B cells, and NK cells, indicating that JKAP may be involved in several important biological processes (9, 10). JKAP is disclosed to mainly regulate mitogen-activated protein kinases (MAPK) signaling pathway, in which it specifically activates c-Jun N-terminal kinases (JNK) pathway but not extracellular-signal-related kinases pathway or p38 MAPK pathway in mammalian cells, and JKAP-knockout murine embryonic stem cells lack JNK activation in response to stimulation by TNF-α or transforming growth factor-β (10, 11). Recently, it has been reported that JKAP activates T-cell receptor (TCR) signaling via directly inactivating Lck, and JKAP-knockout T cells illuminate improved cell proliferation and abundant inflammation cytokines’ expressions (12). And JKAP-knockout mice develop exacerbated inflammation and autoimmunity compared with wide-type mice and are more likely to present with experimental autoimmune encephalomyelitis (EAE), resulting from the enhanced T-cell-mediated adaptive immune responses with higher secretion of pro-inflammation cytokines including IFN-γ, IL-17, and IL-4 (12). Besides, JKAP expression has been demonstrated to be decreased in peripheral blood T cells from systemic lupus erythematosus (SLE) and be correlated with the clinical activity of this autoimmune disease (13). These studies imply that JKAP may be a key factor in regulating inflammation and immune responses, which might play an important role in the development and progress of IBD as well.

Although great advances in the understanding of IBD have been achieved in recent decades, the diagnosis of IBD is still dependent on the endoscopy examination and histological finding, which are invasive and expensive, and the treatment of IBD is not satisfactory for every patient, despite the development of TNF inhibitors such as infliximab (IFX), which greatly improves the outcome in IBD patients (14–19). Therefore, finding new potential biomarkers, which are useful in the diagnosis and individualized treatment of IBD, is worth studying. In the current study, we aimed to investigate the expression of JKAP in the intestinal mucosa of patients with IBD and its potential role in inhibiting inflammatory responses in IBD.

## Materials and Methods

### Patients

All the CD and UC patients were consecutively recruited at the Department of Gastroenterology, Zhongnan Hospital of Wuhan University, from February 2015 to July 2016. Intestinal biopsies were collected during endoscopy from 25 healthy volunteers [healthy controls (HCs)], 18 active CD patients, 22 CD patients with remission, 19 active UC patients, and 22 UC patients with remission. Paired inflamed and unaffected intestinal biopsies were obtained from the same patients with active CD and UC (Table 1). In the meanwhile, EDTA anti-coagulated blood samples were obtained from 10 HCs, 10 active CD patients, and 10 active UC patients to isolate CD4^+^ T cells for further experiments.

**Table 1:** The baseline demographic and clinical characteristics of patients with IBD.

Parameters	CD		UC	
	A-CD	R-CD	A-UC	R-UC
Number	18	22	19	22
Age (years)	31.9 ± 9.3	35.1 ± 8.2	37.8 ± 11.5	38.1 ± 9.9
Gender (male/female)	10/8	9/13	10/9	10/12
CRP (mg/L)	46.83 (32.72, 66.10)	17.04 (14.93, 30.60)	48.55 (34.94,69.80)	24.12 (18.96, 31.94)
ESR(mm/h)	44.94 (38.48, 58.20)	10.96 (16.45, 21.95)	41.24 (29.47, 51.03)	16.41 (11.28, 19.49)
CDAI for CD	183 (161.5, 227.75)	113 (91.25, 139.25)		
Mayo for UC			5 (4, 9)	1 (1,2)

*Data were presented as mean ± SD, median (25th–75th), or count*.

*CD, Crohn’s disease; A-CD, active Crohn’s disease; R-CD, Crohn’s disease with remission; UC, ulcerative colitis; A-UC, active ulcerative colitis; R-UC, ulcerative colitis with remission; CRP, C-reactive protein; ESR, erythrocyte sedimentation rate; CDAI, Crohn’s disease activity index*.

All the patients were diagnosed according to the clinical characteristics, radiological findings, endoscopic examination, and histological results. The clinical severity of diseases was measured on the basis of Crohn’s disease activity index (CDAI) for CD and Mayo index for UC. Furthermore, the levels of C-reactive protein (CRP) and erythrocyte sedimentation rate (ESR) of these patients were collected. This study was approved by the Ethnics Review Board of Zhongnan Hospital of Wuhan University, and all subjects (healthy volunteers and IBD patients) gave written informed consent. This study was conducted in accordance with the Declaration of Helsinki.

### Analysis of mRNA Expression

Total RNA from tissues or cells was extracted using the Trizol reagent (Invitrogen, UAS) and, then, subjected to reverse transcription with the 5× All-In-One RT MasterMix kit (Applied Biological Materials, Canada). Subsequently, qRT-PCR was performed with a SYPR Green PCR kit (TaKaRa, Japan), and the levels of mRNA were analyzed using the 2^−ΔΔCt^ method with GAPDH as internal reference. The primers used in this study are listed in Table 2.

**Table 2:** The sequence of primers.

Gene	Species	Sequence (5′–3′)	Sequence (5′–3′)
JNK pathway-associated phosphatase	Human	AGCAGCGGATTCACCATCTC	TGATGTATGCGATCACCAGTGT
Gapdh	Human	GGAGCGAGATCCCTCCAAAAT	GGCTGTTGTCATACTTCTCATGG
Interferon-γ	Human	TCGGTAACTGACTTGAATGTCCA	TCGCTTCCCTGTTTTAGCTGC
Tumor necrosis factor-α	Human	CCTCTCTCTAATCAGCCCTCTG	GAGGACCTGGGAGTAGATGAG
Interleukin (IL)-17A	Human	TCCCACGAAATCCAGGATGC	GGATGTTCAGGTTGACCATCAC
IL-10	Human	AGGGCACCCAGTCTGAGAACA	CGGCCTTGCTCTTGTTTTCAC
T-bet	Human	GGTTGCGGAGACATGCTGA	GTAGGCGTAGGCTCCAAGG
RORC	Human	GTGGGGACAAGTCGTCTGG	AGTGCTGGCATCGGTTTCG

### Immunoblotting Analysis

The protein level of JKAP was measured by immunoblotting. Briefly, total proteins were extracted from cells or tissues, fractioned on SDS-PAGE, and transferred to PVDF membranes. After blocking, anti-human JKAP antibody (Abcam, USA) and anti-human-GAPDH antibody (Abcam, USA) were incubated with the membranes overnight at 4°C. Then, the membranes were incubated with the secondary antibody (Abcam, USA) for 1 h at room temperature after washing. ImageJ software (Java, USA) was used to determine the density of immunoblotting results, and relative density of target protein was normalized by GAPDH density as a ratio.

### IFX Treatment for Patients with CD

Twenty-five active CD patients (8 cases from 18 active CD patients in original cohort of this study and 17 cases from the history cohort) were infused with IFX (5 mg/kg) at weeks 0, 2, and 6 according to the manufacturer’s instructions (20), and intestinal mucosa was collected at weeks 0 and 12 and stored at −80°C. JKAP protein and mRNA expression were detected. The clinical efficacy of IFX was assessed at week 12 after initial infusion of IFX in this study, and we divided these patients into two groups (response and failure) according to changes in CDAI scores. A decrease in the CDAI score ≥70 points after IFX treatment was asked in response group, while patients with the change of the CDAI <70 points were put into failure group (21).

### Isolation of Peripheral Blood CD4^+^ T Cells

Peripheral blood mononuclear cells were isolated from EDTA anticoagulated blood samples obtained from 10 HCs, 10 active CD patients, and 10 active UC patients by density centrifugation using Ficoll-Paque™ Plus (GE Healthcare Bio-Science, USA). Subsequently, peripheral blood CD4^+^ T cells were isolated using Anti-Human CD4 Particles (BD Biosciences, USA). The purity of peripheral blood CD4^+^ T cells was >95% assessed by flow cytometry.

### CD4^+^ T-Cell Transduction

Lentivirus carrying green-fluorescent protein was used in our study, and Lentivirus-encoding JKAP (LV-JKAP), the empty vector (LV-scramble), or LV-siRNA (LV-anti-JKAP) was transduced into peripheral blood CD4^+^ T cells according to the manufacturer’s protocol. First, peripheral blood CD4^+^ T cells were stimulated with anti-CD3 (5 mg/mL) and anti-CD28 (2 mg/mL) for 24 h. Second, lentivirus (multiplicity of infection, MOI = 100) was incubated with those CD4^+^ T cells overnight. After washing, those transduced cells were cultured with anti-CD3 (5 mg/mL) and anti-CD28 (2 mg/mL) for another 72 h. Otherwise, CD4^+^ T cells without LV labeling as “medium” were used as negative control in this study.

### Analysis of CD4^+^ T-Cell Activation

Transduced cells were harvested after 72 h, followed by staining with anti-human-CD25 or anti-human-CD69 (BD Biosciences, USA). Then, the percentages of CD25^+^ cells and CD69^+^ cells were analyzed using a BD FACSCanto II.

### CD4^+^ T-Cell Proliferation Assay

CD4^+^ T-cell proliferation assay was conducted according to manufacturer’s protocol. Briefly, those transduced cells were labeled with CFSE (5 µM) and cultured with anti-CD3 (5 mg/mL) and anti-CD28 (2 mg/mL) for another 72 h. The CFSE signal was then measured by flow cytometry.

### Statistical Analysis

Data of this study were expressed as mean ± SD or medium (IQR), and statistical analysis was performed using SPSS Version 19.0 (SPSS, Chicago, IL, USA). Paired *t* test and unpaired *t* test were performed to measure the differences between groups, receiver operator characteristic (ROC) curve was drawn to determine the diagnostic value of JKAP expression for CD and UC, respectively, and Spearman’s correlation was used to determine the correlation of JKAP expression with clinical activity and the levels of inflammatory cytokines in IBD patients. Statistical significance was set as *P < 0.05, **P < 0.01, and ***P < 0.001.

## Results

### Patient Characteristics

The baseline demographic and clinical characteristics of the enrolled patients with IBD are listed in Table 1. Among 81 patients, there were 40 CD patients (18 active CD patients and 22 CD patients with remission) and 41 UC patients (19 active UC patients and 22 UC patients with remission). The average ages of enrolled patients were 31.9 ± 9.3 years (10 males and 8 females), 35.1 ± 8.2 years (9 males and 13 females), 37.8 ± 11.5 years (10 males and 9 females), and 38.1 ± 9.9 years (10 males and 12 females) for active CD, CD patients with remission, active UC, and UC patients with remission, respectively. As to clinical indexes, the medium levels of CRP of active CD, CD patients with remission, active UC, and UC patients with remission were 46.83 (32.72, 66.10), 17.04 (14.93, 30.60), 48.55 (34.94, 69.80), and 24.12 (18.96, 31.94), while the levels of ERS were 44.94 (38.48, 58.20), 10.96 (16.45, 21.95), 41.24 (29.47, 51.03), and 16.41 (11.28, 19.49), respectively.

### Expression of JKAP in Intestinal Mucosa of IBD Patients

**Figure 1:**
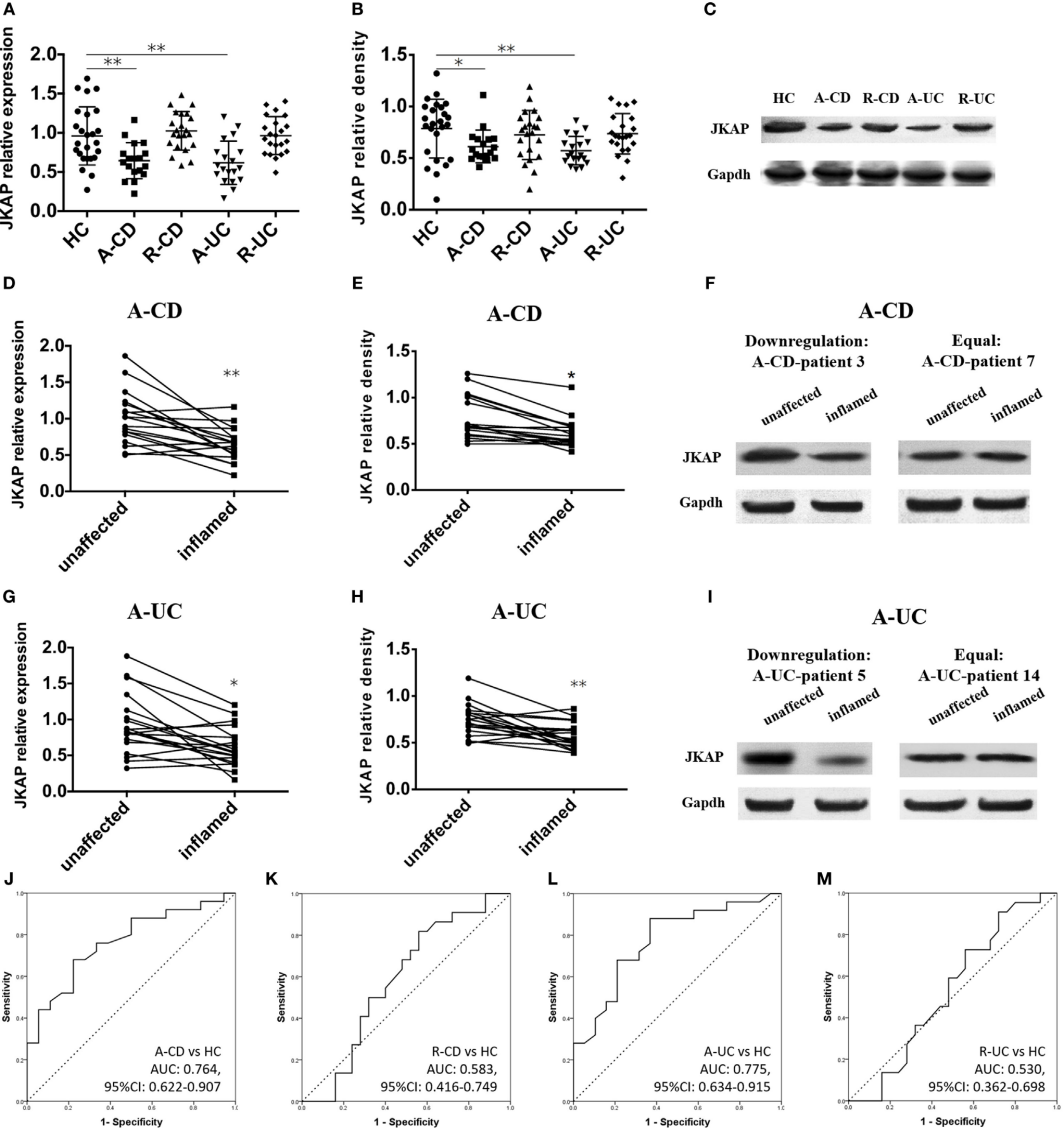
Expression of JNK pathway-associated phosphatase (JKAP) in intestinal mucosa of inflammatory bowel disease (IBD) patients and healthy control (HC). JKAP mRNA expression was decreased in both active Crohn’s disease (A-CD) patients and active ulcerative colitis (A-UC) patients than in HCs, while no difference was found of HCs with Crohn’s disease with remission (R-CD) patients and ulcerative colitis with remission (R-UC) patients **(A)**. Similar trends were observed in JKAP protein expression **(B)** and **(C)** presented JKAP protein expression of representative patients. Both JKAP mRNA expression **(D)** and protein expression **(E)** were decreased in inflamed mucosa than in unaffected mucosa in A-CD patients, and they were decreased in A-UC patients **(G,H)**. **(F)** Two representative A-CD patients; the left showed that JKAP protein expression was dramatically decreased in inflamed mucosa than in unaffected mucosa in A-CD-patient 3, while the right showed JKAP protein expression remained the same in inflamed mucosa than in unaffected mucosa in A-CD-patient 7. **(I)** Two representative A-UC patients; the left showed JKAP protein expression was dramatically decreased in inflamed mucosa than in unaffected mucosa in A-UC-patient 5, while the right showed JKAP protein expression remained the same in A-UC-patient 14. Furthermore, receiver operator characteristic (ROC) curves displayed that JKAP mRNA expression possessed moderate diagnostic value for A-CD **(J)** and A-UC **(L)** from HC, while no diagnostic value for R-CD **(K)** or R-UC **(M)** from HC was discovered. *t* test was used to determine the difference between two groups, while paired *t* test was used to detect the difference between paired inflamed and unaffected mucosa. ROC curves were drawn to analyze the diagnostic value of JKAP for IBD. Statistical significance was set as *P < 0.05 and **P < 0.01.

Since JKAP has been found to regulate TCR signaling and be involved in the pathogenesis of SLE, we then sought to assess the expression of JKAP in inflamed intestinal mucosa of IBD patients. We found that both mRNA (Figure 1A) and protein (Figures 1B,C) expressions of JKAP were significantly down-regulated in inflamed mucosa of active CD and UC compared to that from HCs, while there was no statistical difference between HCs and CD as well as UC patients with remission. In the meanwhile, paired inflamed and unaffected mucosa from the same active CD and UC patients were also collected for analyzing JKAP expression and we observed that JKAP mRNA expression was significantly decreased in the inflamed intestinal mucosa compared with the unaffected mucosa from the same patients with active CD and UC (Figures 1D,G). Consistently, protein expression of JKAP was also found to be decreased in the inflamed intestinal mucosa compared with the unaffected mucosa from the same active CD and UC patients (Figures 1E,F,H,I). Collectively, these data indicated that JKAP expression was decreased in inflamed intestinal mucosa of active IBD patients, and JKAP may play an important role in the pathogenesis of IBD. In addition, ROC curves were performed to evaluate the diagnostic value of JKAP mRNA expression for IBD, which disclosed that JKAP had a good diagnostic value for A-CD (Figure 1J) and active ulcerative colitis (Figure 1L), but no diagnostic power for CD with remission (R-CD) (Figure 1K) and ulcerative colitis with remission (Figure 1M) was discovered.

### Correlation of JKAP Expression with Clinical Activity of IBD Patients

**Figure 2:**
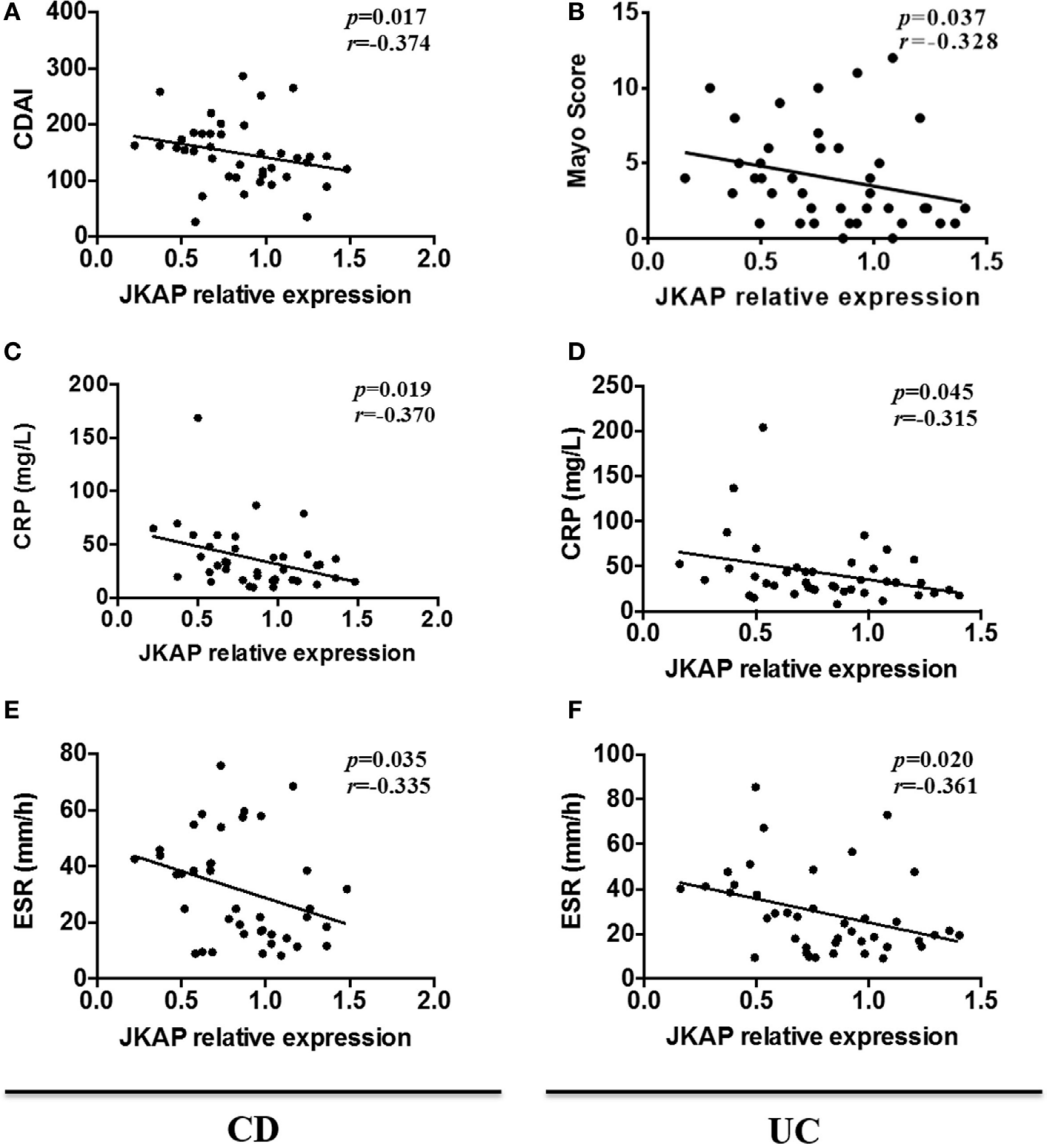
Correlation of JNK pathway-associated phosphatase (JKAP) expression with the clinical activity of inflammatory bowel disease patients. JKAP mRNA expression was negatively correlated with disease activity in both Crohn’s disease (CD) and ulcerative colitis (UC) patients: in CD patients (active Crohn’s disease and Crohn’s disease with remission), mucosa JKAP mRNA expression was negatively correlated with Crohn’s disease activity index score **(A)**, C-reactive protein (CRP) level **(C)**, and erythrocyte sedimentation rate (ESR) level **(E)**; while in UC patients (active ulcerative colitis and ulcerative colitis with remission), mucosa JKAP mRNA expression was also negatively associated with Mayo score **(B)**, CRP level **(D)**, and ESR level **(F)**. Spearman test was used to determine the correlation between two parameters.

Considering that CDAI, Mayo index for UC, CRP, and ESR are commonly used to assess the clinical activity in IBD patients (22–24), we next determined the correlation of JKAP expression with CDAI, Mayo index, CRP, and ESR, respectively. As shown in Figures 2A,B, JKAP expression in intestinal mucosa was significantly negatively correlated with CDAI in CD patients and Mayo index in UC patients. Moreover, it was observed that JKAP expression in intestinal mucosa of CD and UC patients was negatively correlated with CRP (Figures 2C,D). A negative correlation between JKAP expression and ESR was also found in CD and UC patients (Figures 2E,F). Taken together, these data showed that JKAP expression in intestinal mucosa was negatively correlated with clinical activity in IBD patients.

### Correlation of JKAP Expression with Inflammatory Cytokine Levels in IBD Patients

**Figure 3:**
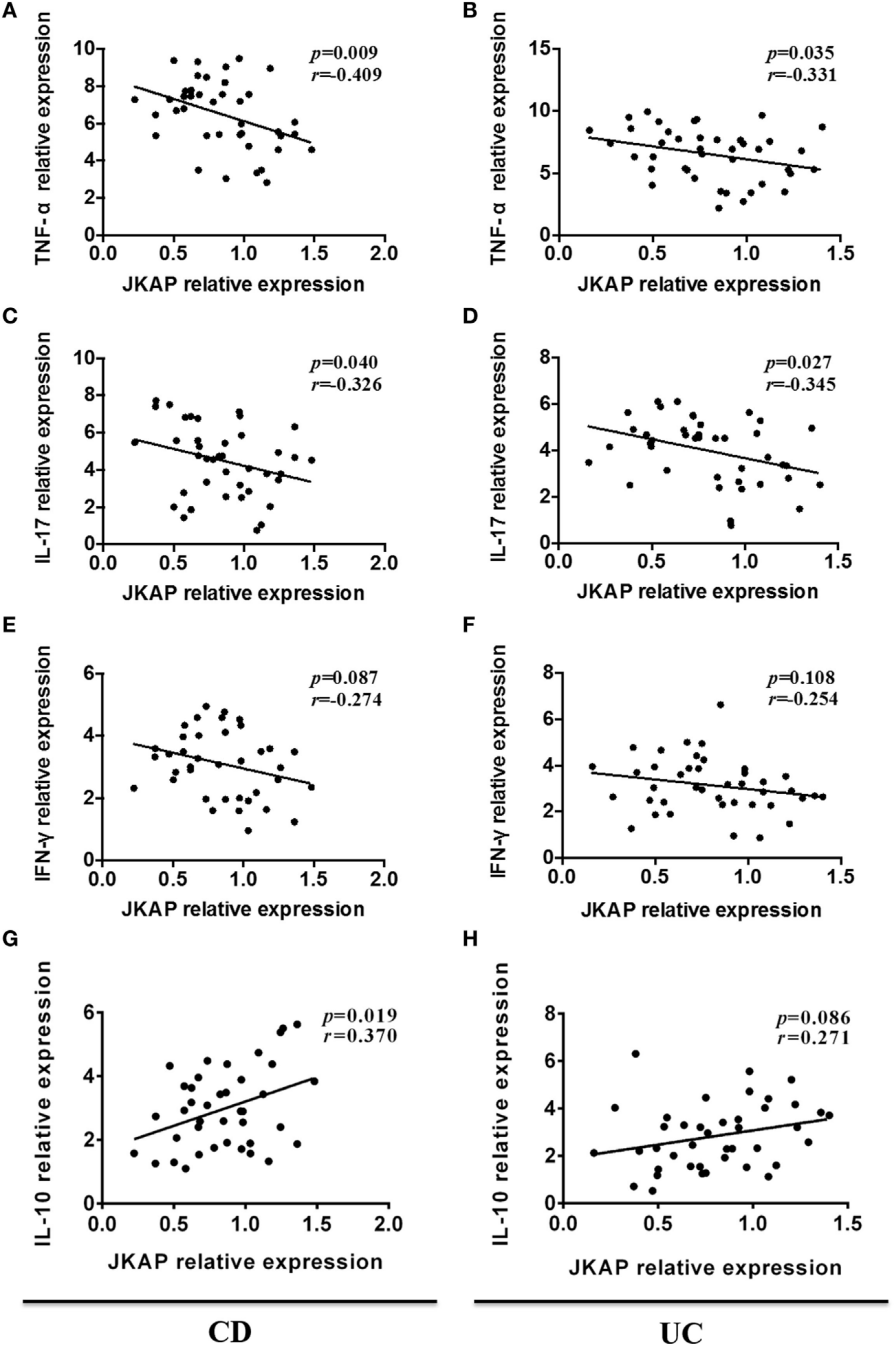
Correlation of JNK pathway-associated phosphatase (JKAP) expression with inflammatory cytokines levels in inflammatory bowel disease patients. In Crohn’s disease patients (active Crohn’s disease and Crohn’s disease with remission), mucosa JKAP mRNA expression was observed to be negatively correlated with tumor necrosis factor (TNF)-α **(A)** and interleukin (IL)-17 **(C)** mRNA expressions and positively correlated with IL-10 mRNA **(G)** expression. No association between JKAP and interferon (IFN)-γ mRNA expressions was found, but a negatively correlated trend was discovered **(E)**. In ulcerative colitis patients (active ulcerative colitis and ulcerative colitis with remission), JKAP mRNA expression was also reversely correlated with TNF-α **(B)** and IL-17 **(D)** mRNA expressions. No correlation of JKAP mRNA expression with IFN-γ **(F)** and IL-10 **(H)** mRNA expressions was disclosed. Spearman test was used to determine the correlation between two parameters.

Since previous studies have demonstrated that several inflammatory cytokines were dysregulated in IBD patients, and serum levels of IFN-γ, IL-17, and TNF-α are significantly up-regulated, while IL-10 is decreased in JKAP-knockout mice, we next asked whether there was a correlation between JKAP mRNA expression and the levels of IFN-γ, IL-17, TNF-α, and IL-10 in intestinal mucosa from IBD patients. As shown in Figures 3A,B, the mRNA expression of TNF-α was negatively correlated with JKAP mRNA expression in intestinal mucosa from CD patients and UC patients. The level of IL-17 was also found to be negatively correlated with JKAP expression in intestinal mucosa from CD patients and UC patients (Figures 3C,D). However, no statistical correlation between IFN-γ and JKAP expressions in intestinal mucosa from CD patients and UC patients were observed (Figures 3E,F). As to IL-10 mRNA level, we found that it was positively associated with JKAP mRNA expression in CD patients (Figure 3G), while in UC patients, no correlation was observed (Figure 3H). Collectively, these data demonstrated that JKAP expression was negatively correlated with IL-17 and TNF-α in intestinal mucosa from IBD (both CD and UC) patients and positively associated with IL-10 in only CD patients.

### The Difference Expression of JKAP before and after IFX Treatment

**Figure 4:**
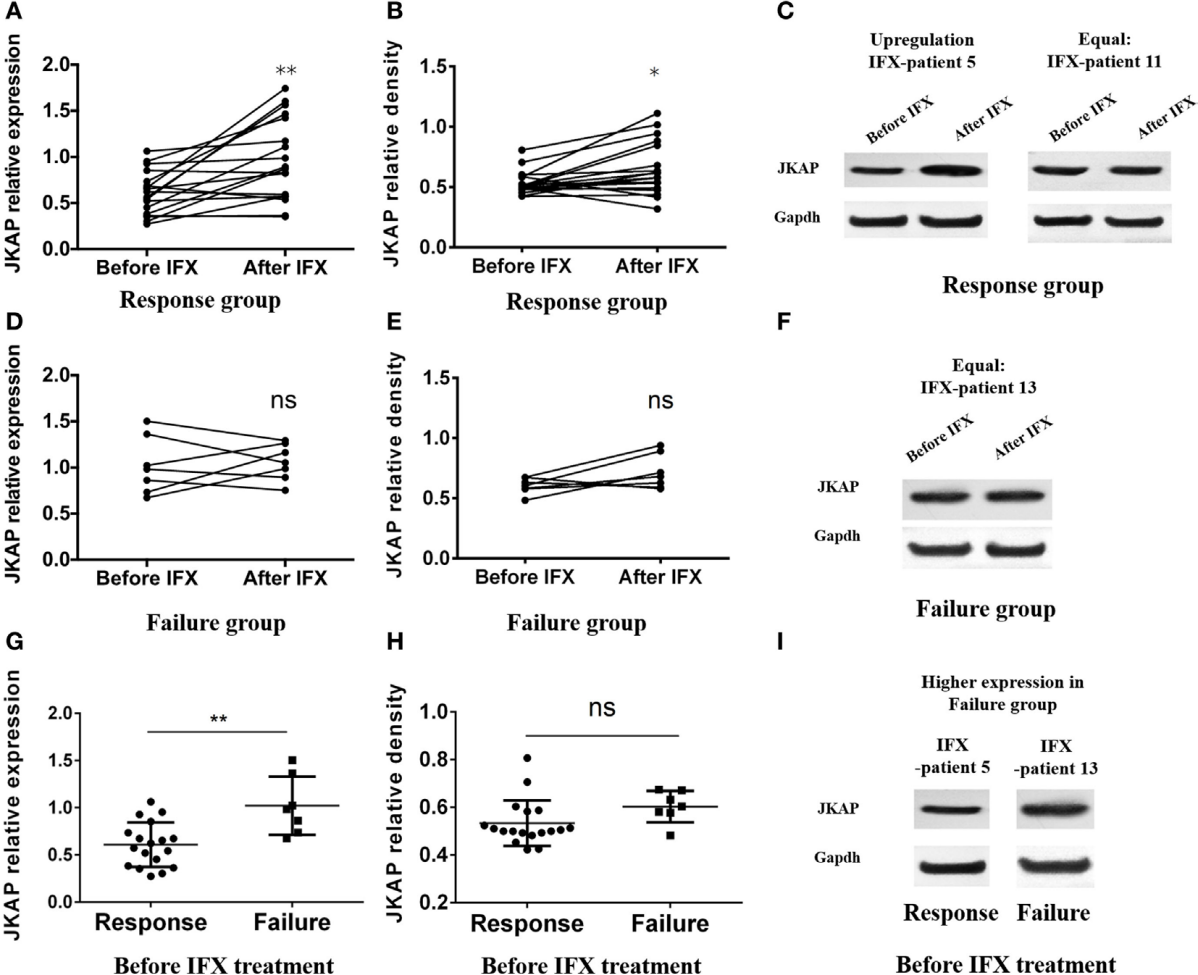
The difference expression of JNK pathway-associated phosphatase (JKAP) before and after infliximab (IFX) treatment in Crohn’s disease patients. Both JKAP mRNA **(A)** and protein expressions **(B)** were increased after IFX treatment in the response group, while no difference was discovered in either JKAP mRNA expression **(D)** or protein expression **(E)** before and after IFX treatment in the failure group. **(C)** Two representative patients; the left showed that JKAP protein expression was dramatically raised after IFX treatment in IFX-patient 5, while the right showed that JKAP protein expression stayed the same after IFX treatment in IFX-patient 11. **(F)** One representative patient whose JKAP protein expression remained equal after IFX treatment (IFX-patient 13). As to baseline JKAP, we found that baseline JKAP mRNA expression was increased in the failure group than in the response group **(G)**, while JKAP protein was numerically raised in the failure group than in the response group but without statistical significance **(H)**. **(I)** Two representative patients, which showed that JKAP protein expression was higher in failure patient (IFX-patient 13) than in response patient (IFX-patient 5). Paired *t* test was used to determine the difference of JKAP mRNA and protein expressions before and after IFX treatment. *t* test was used to detect the difference of baseline JKAP mRNA and protein expressions between the response and failure groups. Statistical significance was set as *P < 0.05 and **P < 0.01.

Infliximab, a kind of anti-TNF-α mAb, is usually used to treat IBD patients and has been widely used for CD patients in our department. In last part, we found that JKAP expression in intestinal mucosa was negatively correlated with clinical activity in IBD patients and we then investigated whether IFX treatment could up-regulate JKAP expression in IBD patients. Due to that the indication of IFX is limited to CD but not UC in China, we only included 25 patients with active CD who were treated by IFX at weeks 0, 2, and 6 as indicated, and JKAP expressions were measured in intestinal mucosa of these patients at weeks 0 and 12. After treatment, 18 cases (72%) achieved clinical response (response group), while 7 cases (28%) failed (failure group) at week 12. In response group, IFX dramatically up-regulated both JKAP mRNA expression (Figure 4) and protein expression (Figures 4B,C) in intestinal mucosa, but no statistical change of JKAP expression in intestinal mucosa was observed in the failure group (Figures 4D–F). Collectively, these data indicated that IFX treatment could up-regulate JKAP expression in intestinal mucosa from CD patients in response group. Otherwise, difference of baseline JKAP expression between response group and failure group was analyzed and we found that JKAP mRNA expression was elevated in response group compared to failure group (Figure 4G), while immunoblotting results illuminated that baseline JKAP protein expression was numerically higher in the failure group but without significance (Figures 4H,I). These indicated that JKAP expression might have value in predicting clinical response to IFX treatment.

### The Functions of JKAP on CD4^+^ T Cells in IBD

**Figure 5:**
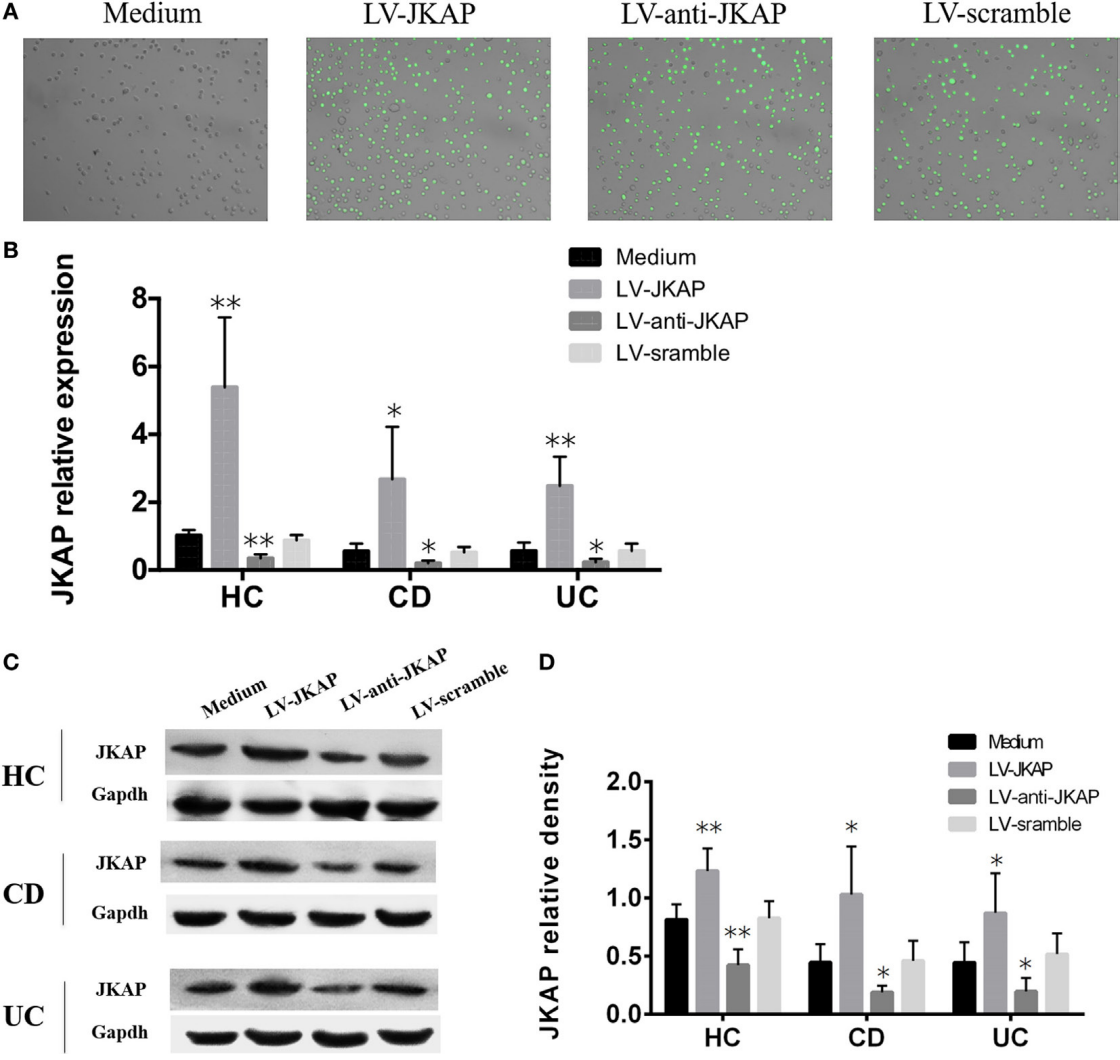
Transduction efficiency of lentivirus into CD4^+^ T cells. Peripheral blood CD4^+^ T cells were transduced with empty vector (LV-scramble), JNK pathway-associated phosphatase (JKAP) lentivirus (LV-JKAP), and si-JKAP lentivirus (LV-anti-JKAP), and CD4^+^ T cells without LV labeling (medium) were used as negative controls. **(A)** Transduction efficiency of each group was as follows: LV-JKAP: 91 ± 2%, LV-anti-JKAP: 92 ± 1%, and LV-scramble: 91 ± 3%. Both JKAP mRNA **(B)** and protein **(D)** expressions were increased in the LV-JKAP group and decreased in the LV-anti-JKAP group from healthy controls (HCs) and Crohn’s disease (CD) and ulcerative colitis (UC) patients. **(C)** Representative samples that LV-JKAP increased the JKAP protein expression and LV-anti-JKAP reduced it in HC and CD and UC patients. *t* test was used to determine the difference between two groups. Statistical significance was set as *P < 0.05 and **P < 0.01.

**Figure 6:**
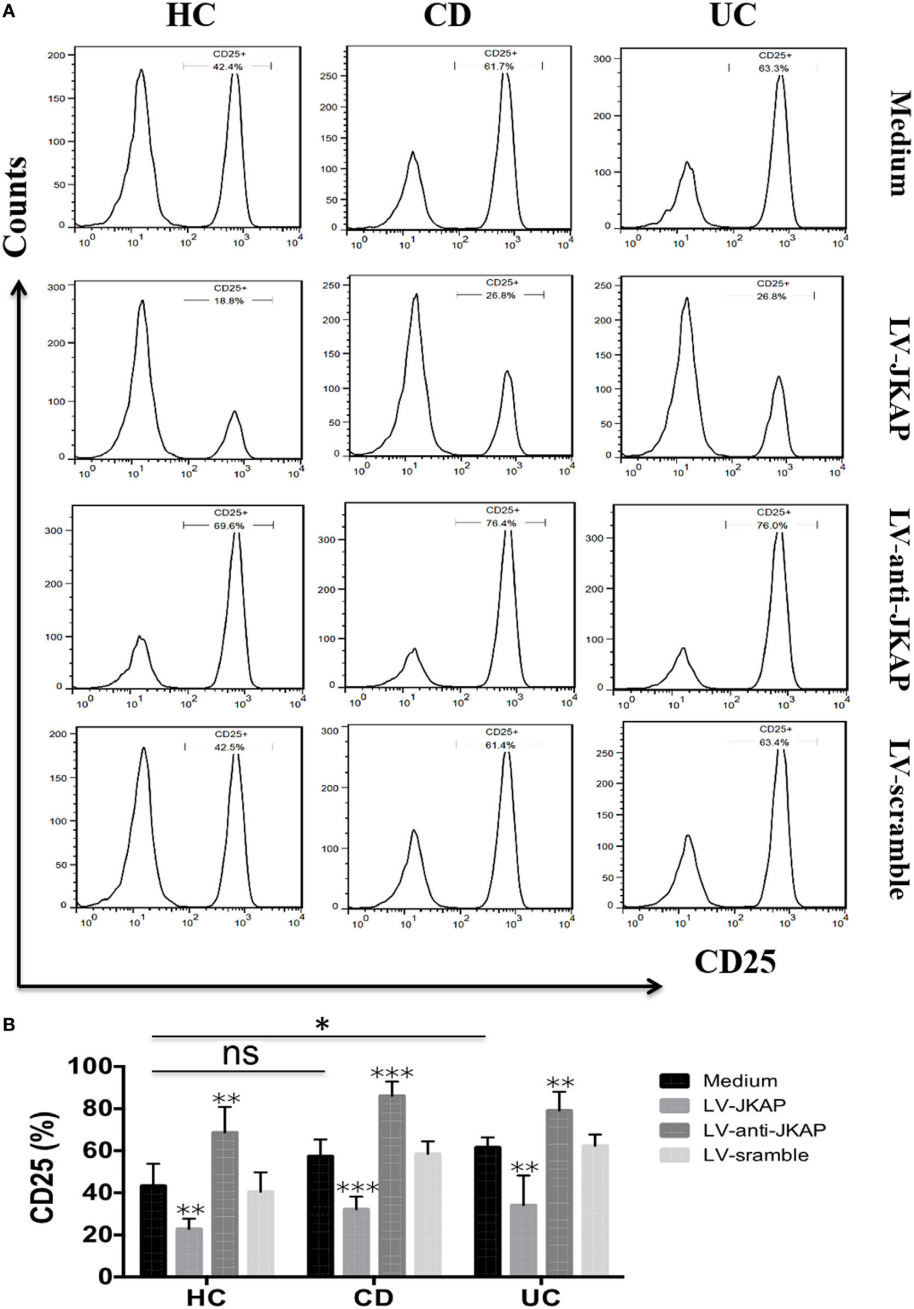
JNK pathway-associated phosphatase (JKAP) inhibits the activation of CD4^+^ T cell by the determination of CD25^+^ cells in inflammatory bowel disease. **(A)** Results of CD25^+^ cells in medium, LV-JKAP, LV-anti-JKAP, and LV-scramble groups from representative healthy controls (HCs) and Crohn’s disease (CD) and ulcerative colitis (UC) patients. **(B)** CD25^+^ cells were decreased in HC-medium group than in the UC-medium group but were the same between the HC-medium and CD-medium groups. LV-JKAP greatly reduced the percentage of CD25^+^ cells in HC and CD and UC patients than in controls, and LV-anti-JKAP remarkably increased the percentage of CD25^+^ cells in HC and CD and UC patients than in controls. *t* test was used to determine the difference between two groups. Statistical significance was set as *P < 0.05, **P < 0.01, and ***P < 0.001.

**Figure 7:**
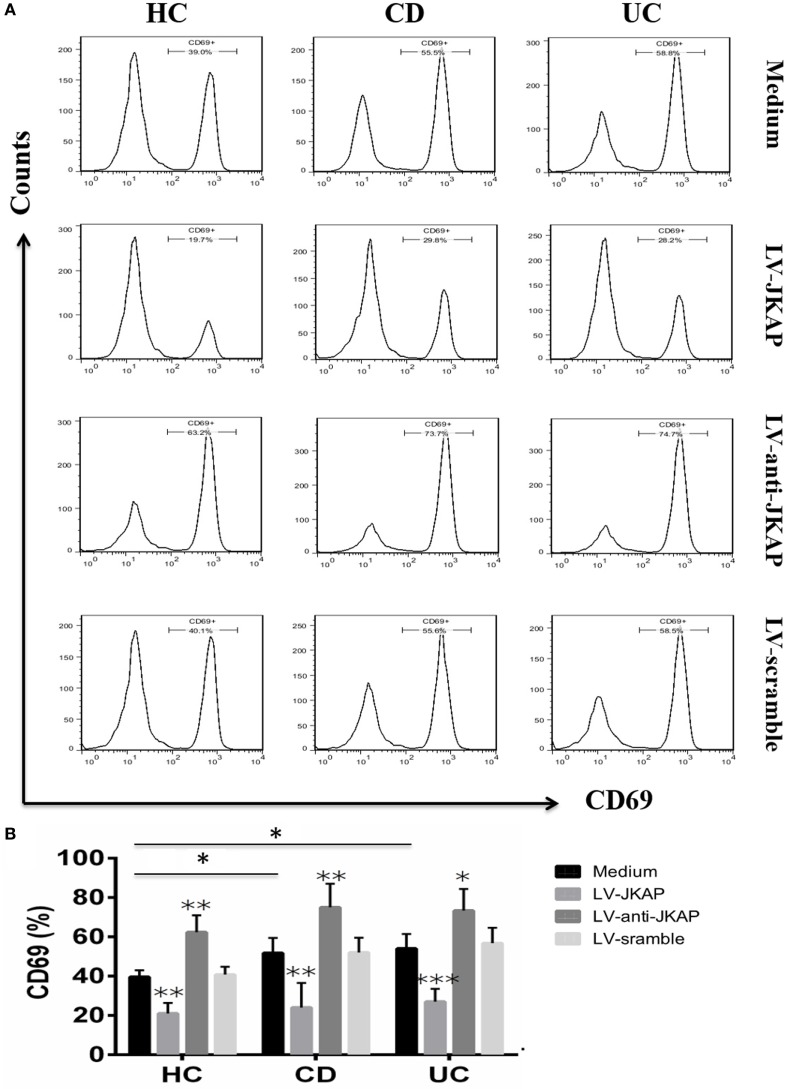
JNK pathway-associated phosphatase (JKAP) inhibits the activation of CD4^+^ T cell by the determination of CD69^+^ cells in inflammatory bowel disease. **(A)** Results of CD69^+^ cells in medium, LV-JKAP, LV-anti-JKAP, and LV-scramble groups from representative healthy controls (HCs) and Crohn’s disease (CD) and ulcerative colitis (UC) patients. **(B)** CD69^+^ cells were decreased in the HC-medium group than in the CD-medium and UC-medium groups, LV-JKAP decreased the percentage of CD69^+^ cells in HC and CD and UC patients than in controls, and LV-anti-JKAP remarkably elevated the percentage of CD69^+^ cells in HC and CD and UC patients than in controls. *t* test was used to determine the difference between two groups. Statistical significance was set as *P < 0.05, **P < 0.01, and ***P < 0.001.

**Figure 8:**
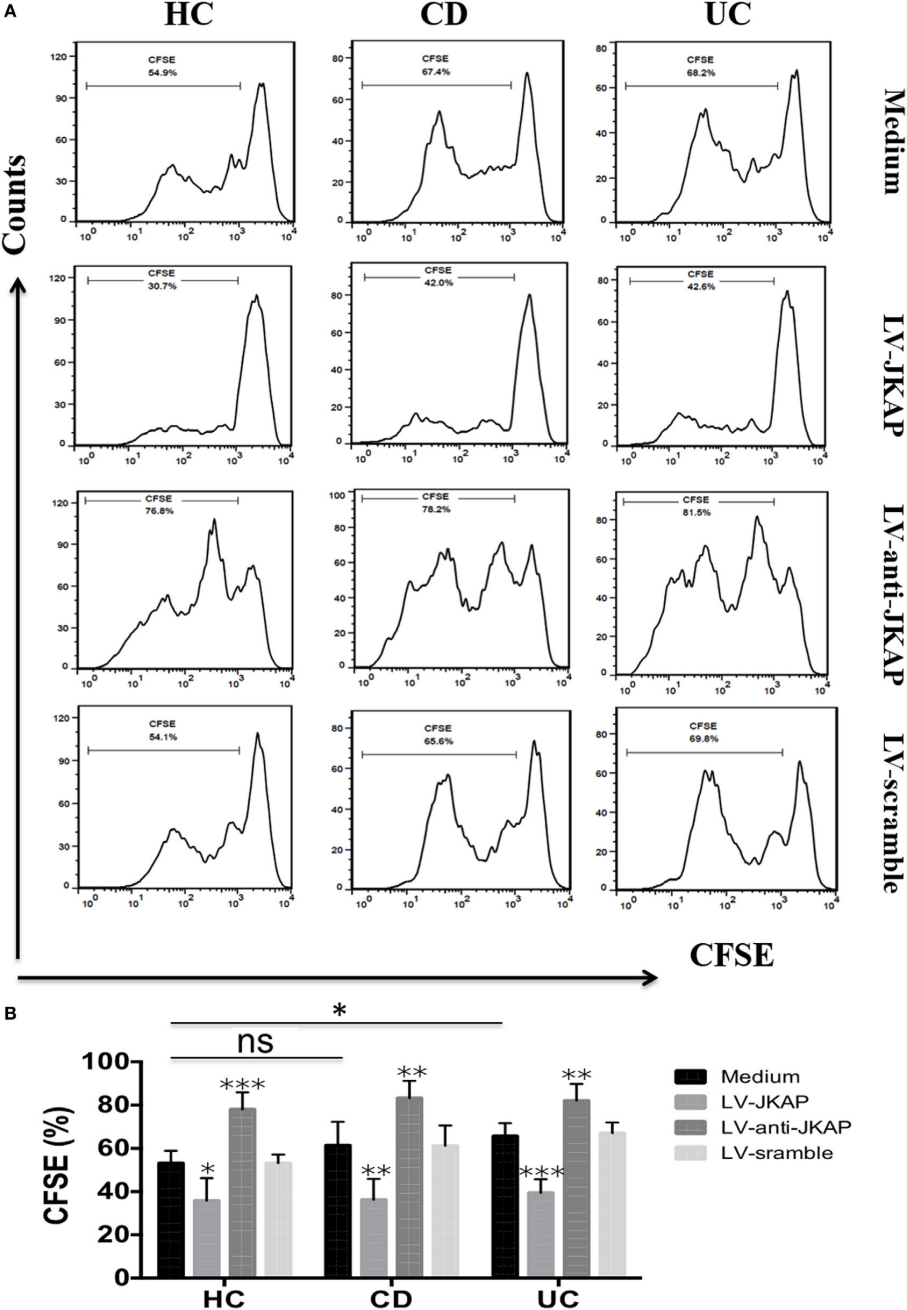
JNK pathway-associated phosphatase (JKAP) inhibits the proliferation of CD4^+^ T cells in inflammatory bowel disease. **(A)** Results of cell divisions in the medium, LV-JKAP, LV-anti-JKAP, and LV-scramble groups from representative healthy controls (HCs) and Crohn’s disease (CD) and ulcerative colitis (UC) patients. **(B)** Cell divisions were decreased in the HC-medium group than in the UC-medium group but not in the CD-medium group. **(B)** LV-JKAP decreased the cell divisions in HC and CD and UC patients than in controls, and LV-anti-JKAP remarkably increased the cell divisions in HC and CD and UC patients than in controls. *t* test was used to determine the difference between two groups. Statistical significance was set as *P < 0.05, **P < 0.01, and ***P < 0.001.

Previous study has reported that JKAP deficiency could enhance T-cell activation in EAE, and we next investigated whether JKAP affected CD4^+^ T-cell activation in IBD. To this end, peripheral blood CD4^+^ T cells isolated from 10 HCs and 10 active CD and 10 active UC patients were individually transduced with empty vector (LV-scramble), JKAP lentivirus (LV-JKAP), si-JKAP lentivirus (LV-anti-JKAP), respectively, and CD4^+^ T cells without LV labeling (medium) were used as negative control. CD4^+^ T cells were then stimulated with anti-CD3 and anti-CD28 for 72 h. First, transduction efficiency was evaluated by fluorography, qRT-PCR, and Immunoblotting. These transduced cells were observed under a microscope, and we found that the transduction efficiency was more than 90% (LV-JKAP: 91 ± 2%, LV-anti-JKAP: 92 ± 1%, and LV-scramble: 91 ± 3% as presented in Figure 5A). Besides, we found that both JKAP mRNA expression and protein expression were significantly increased in LV-JKAP-transduced cells, while it was decreased in LV-anti-JKAP-transduced cells compared to both negative controls and LV-Scramble-transduced cells (Figures 5B–D). The percentages of CD25^+^ cells and CD69^+^ cells were analyzed by cytometry. As shown in Figures 6A,B, the percentages of CD25^+^ cells were significantly decreased in IBD CD4^+^ T cells after transduction with LV-JKAP. Meanwhile, the percentages of CD25^+^ cells were increased in LV-anti-JKAP-transduced IBD CD4^+^ T cells compared to controls (Figures 6A,B). And the similar pattern was found in CD69^+^ cells (Figures 7A,B). Besides, we also examined the proliferation of these cells, and we found that CD4^+^ T cells transduced with LV-JKAP illustrated lesser cell divisions, while CD4^+^ T cells transduced with LV-anti-JKAP underwent more cell divisions compared to controls (Figures 8A,B).

**Figure 9:**
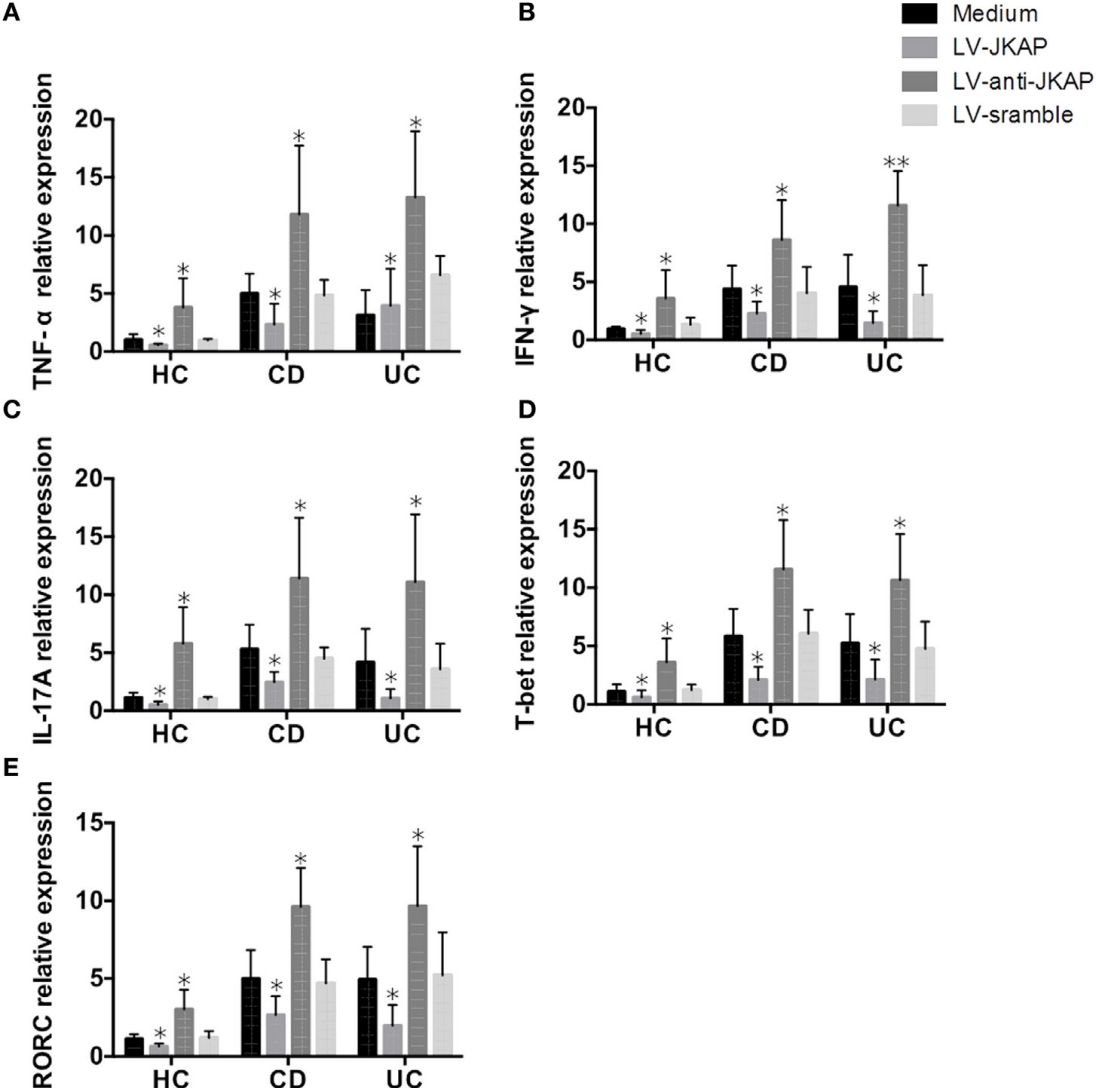
JNK pathway-associated phosphatase (JKAP) inhibits Th1/Th17 differentiation in inflammatory bowel disease. After transduction of LV-JKAP, tumor necrosis factor (TNF)-α **(A)**, interferon (IFN)-γ **(B)**, interleukin (IL)-17A **(C)**, T-bet **(D)**, and RORC **(E)** were decreased compared to controls in healthy controls (HCs) and Crohn’s disease (CD) and ulcerative colitis (UC) patients. While after transduction of LV-anti-JKAP, TNF-α **(A)**, IFN-γ **(B)**, IL-17A **(C)**, T-bet **(D)**, and RORC **(E)** were elevated compared to controls in HCs and CD and UC patients.

Since Th1 and Th17 cells are critical for pathogenesis of IBD, and JKAP has been found to regulate the levels of IFN-γ and IL-17 in EAE, we then asked whether JKAP regulates CD4^+^ T-cell differentiation in IBD. To this end, transduced cells were also collected and the mRNA expression of TNF-α, IFN-γ, and IL-17 was analyzed. Expressions of TNF-α, IFN-γ, and IL-17 were significantly down-regulated when JKAP was overexpressed in CD4^+^ T cells from IBD patients, while they were increased in LV-anti-JKAP-transduced IBD CD4^+^ T cells than in controls (Figures 9A–C). Consistently, the mRNA levels of T-bet and RORC were also significantly decreased in IBD CD4^+^ T cells after transduction of JKAP (Figures 9D,E).

Taken together, these data demonstrated that JKAP suppresses the activation and proliferation of CD4^+^ T cells and inhibits Th1 and Th17-cell differentiation in IBD.

## Discussion

Substantial researches have shown that IBD is a chronic disorder affecting gastrointestinal tract and results in poor quality of life with relatively high disability rate (1, 25, 26). Despite many improvements in diagnosis, novel treatment drugs, optimized treatment strategy, and so on, the early diagnosis remains lacking convincing markers and 20–30% patients still present with insufficient efficacy. Thus, novel and promising biomarkers for diagnosis and treatment are deadly needed (19, 27, 28). In this study, we found that (1) JKAP expression was significantly decreased in inflamed intestinal mucosa from active IBD patients and negatively correlated with clinical activity and pro-inflammatory cytokines levels (IL-17 and TNF-α). (2) The level of JKAP was significantly increased in the intestinal mucosa from CD patients after the treatment of IFX in the response group, and baseline JKAP expression might be able to predict clinical response to IFX treatment. (3) JKAP inhibited the activation and proliferation of CD4^+^ T cells and Th1/Th17 differentiation in IBD. Our study provided evidence that JKAP might be a potential marker for the diagnosis and treatment of IBD.

Although great advances in the understanding of IBD have been achieved in recent decades, the diagnosis of IBD is still dependent on the endoscopy examination and histological finding, which are invasive and expensive, and the treatment of IBD is not worked for every patient (14, 15). Therefore, finding new potential biomarkers, which are useful in the diagnosis and individualized treatment of IBD, is worth studying. In this study, we demonstrated that the decreased expression of JKAP in intestinal mucosa contributes to the pathogenesis of IBD, through facilitating CD4^+^ T-cell activation, proliferation, and Th1/Th17-cell differentiation.

We found that JKAP expression was significantly decreased in inflamed intestinal mucosa from active IBD patients and negatively correlated with clinical activity and pro-inflammatory cytokines levels (IL-17 and TNF-α). The level of JKAP was significantly increased in the intestinal mucosa from CD patients after the treatment of IFX in response group. JKAP inhibited the activation and proliferation of CD4^+^ T cells and Th1/Th17 differentiation in IBD. Our study provides evidence that JKAP might be a potential marker for the diagnosis and treatment of IBD.

Dual-specificity phosphatase, a group of cysteine-based protein tyrosine phosphatases, has been reported to participate in several human diseases via dephosphorylation of various substrates, especially cancer and autoimmune diseases (29, 30). Recently, it has been reported that JKAP (DUSP22), as a member of DUSPs, activates TCR signaling and JKAP-knockout mice develop exacerbated autoimmunity through enhancing T-cell immune responses (12). Besides, two recent studies have demonstrated that the level of JKAP was significantly down-regulated in SLE and colorectal cancer patients (13, 31). Considering that both IBD and SLE are belonging to autoimmune diseases and both IBD and colorectal cancer are gastrointestinal diseases, we then examine the expression of JKAP in IBD patients in this study. We found that both JKAP mRNA and protein expressions were significantly decreased in the inflamed mucosa of active CD and UC patients than in HCs, and JKAP mRNA expression could predict active CD status and active UC status from HCs, indicating that JKAP was involved in the pathogenesis of IBD. These might result from that JKAP was an anti-inflammatory gene negatively regulating inflammation and immune response, which was demonstrated in our study as well; thus, lower JKAP was expressed in active IBD patients.

JNK pathway-associated phosphatase expression has been reported to be associated with disease activity and outcome in SLE and colorectal cancer (13, 31), and it is commonly accepted that CDAI, Mayo index for UC, CRP, and ESR are important indicators for clinical disease activity in IBD patients (22–24). To explore the role of JKAP in assessing the disease activity, we examined the correlation of JKAP expression in intestinal mucosa with these indicators. Interestingly, we found that there was a negative correlation of JKAP with CDAI, Mayo index for UC, CRP, and ESR, which indicated that JKAP might a candidate biomarker for evaluating the clinical disease activity in IBD. Besides, IFN-γ, IL-17, TNF-α, and IL-10 mRNA expressions were also detected in mucosa of IBD patients. We found that JKAP mRNA expression was negatively correlated with TNF-α and IL-17 and reversely associated with IL-10 mRNA expression in CD patients. And JKAP mRNA expression was negatively correlated with TNF-α and IL-17 as well in UC patients. These implied the anti-inflammatory role JKAP in IBD, which might be on account of (1) the regulating effect of JKAP on T-cell activation and Th1/Th17-cell differentiation, which disclosed in our study. (2) JKAP suppresses T-cell-mediated adaptive immune responses through regulating JNK, TCR, and IL-6/leukemia inhibitory factor/STAT3 signaling pathways.

Nowadays, IFX, a human monoclonal IgG anti-TNF antibody, has been widely used in alleviating intestinal inflammation in IBD patients (32). IFX has been proven to reduce the inflammation rapidly compared to conventional treatment and achieve both increased clinical remission rate and elevated endoscopic remission rate (16, 17). However, there are still 20–30% of IBD patients lacking response to IFX treatment (18). In our study, we analyzed the JKAP expression in intestinal mucosa before and after IFX treatment from same CD patients and we found that JKAP expression was increased after IFX treatment in the response group, but JKAP expression was not changed significantly after IFX treatment in the failure group. As to different results in these two groups, it might result from that JKAP expression was negatively correlated with CDAI and that we divided patients into the response and failure groups according to the change of CDAI before and after IFX treatment. In addition, we found a lower baseline JKAP mRNA level in the response group than in the failure group, which suggested that the JKAP expression before IFX might have a potential value in predicting clinical response to IFX treatment. The possible reasons were as follows: (1) JKAP expression was negatively correlated with disease activity, such as CDAI score, CRP, ESR, and pro-inflammatory cytokines; thus, the low expression of JKAP represented that patients were in higher disease activity, which provided a higher ranged threshold value for a decrease in CDAI score. Besides, higher pro-inflammatory cytokines expressions might respond better to anti-inflammatory treatment. (2) JKAP expression might have effect on the drug sensitivity to IFX treatment through regulating MAPK, TCR, and IL-6/STAT3 signaling pathways, which needed to be investigated in the future studies. (3) The possibility of JKAP expression with IFX-antibody development might contribute the consequence but still needed to be verified in future researches.

Accumulating evidence has shown that CD4^+^ T cells are critical in mediating the pathogenesis of IBD, but how CD4^+^ T cells are regulated remains unclear. In this study, we examined the function of JKAP in the activation, proliferation, and differentiation of CD4^+^ T cells in IBD. It was demonstrated that the transduction of LV-JKAP into CD4^+^ T cells from patients with IBD inhibited the percentages of CD25^+^ cells, proliferation, and Th1/Th17-cell differentiation. These results were consistent with previous study in mice (12). Many researches focus on the mechanism of JKAP regulation. Interestingly, a reduction in STAT3 luciferase reporter activity is demonstrated in cells transfected with JKAP, while the phosphorylation and activation of STAT3 are enhanced in cells treated with JKAP siRNA (33). Also, a role for JKAP is identified in the inhibition of IL-6-induced STAT3 activation by estradiol (34). And another member of DUSPs, DUSP2, is also down-regulated in IBD patients, and moderate association of with JKAP is discovered (35). The phosphatase DUSP2 also regulates Th17 differentiation by controlling the activity of the transcription activator STAT. It is likely that JKAP may share the same pathway with DUSP2 to regulate T-cell differentiation; however, the possible interacting role between JKAP and DUSP2 needed to be further investigated. Furthermore, JKAP is found to directly bind to and dephosphorylate ERα at Ser^118^ in HEK-293T cells, and conversely, the estradiol-induced phosphorylation of ERα at Ser^118^ is enhanced in cells treated by JKAP siRNA (34). Consequently, JKAP may have more than one physiological substrate and the regulation of specific signaling cascades by this enzyme may be cell type and context specific; thus, it is possible that JKAP could influence the proliferation and differentiation of CD4^+^ T cell from HCs as well as discovered in our study. At last, we also observed that the proliferation and activation of CD4^+^ T cells by JKAP upregulation in IBD patients presented almost like that in HCs, which might result from that JKAP overexpression might greatly suppress the proliferation and activation of CD4^+^ T cells independently through multiple signaling pathways such as MAPK, TCR, and IL-6/STAT3, and its influence was extremely significant on CD4^+^ T cells, which needed to be further verified with larger samples and deeper experiments.

In conclusion, our data indicated that the decreased expression of JKAP in intestinal mucosa contributed to the pathogenesis of IBD, through facilitating CD4^+^ T-cell activation, proliferation, and Th1/Th17-cell differentiation.

## Ethics Statement

This study was carried out in accordance with the recommendations of Ethnics Review Board of Zhongnan Hospital of Wuhan University with written informed consent from all subjects. All subjects gave written informed consent in accordance with the Declaration of Helsinki. The protocol was approved by the “Ethnics Review Board of Zhongnan Hospital of Wuhan University.”

## Author Contributions

RZ and YC conceived and designed the experiments. JL and MC performed the experiments. HW and MH analyzed the data. SL, XW, and QZ contributed to the results discussion and manuscript preparation. All the authors reviewed and approved the final manuscript.

## Conflict of Interest Statement

The authors declare that the research was conducted in the absence of any commercial or financial relationships that could be construed as a potential conflict of interest.

## Abbreviations

IBD, inflammatory bowel disease; CD, Crohn’s disease; UC, ulcerative colitis; TNF, tumor necrosis factor; IL, interleukin; IFN, interferon; DUSPs, dual-specificity phosphatase; JKAP, JNK pathway-associated phosphatase; SLE, systemic lupus erythematosus; IFX, infliximab; CDAI, Crohn’s disease activity index; CRP, C-reactive protein; ESR, erythrocyte sedimentation rate; LV-scramble, the empty vector; LV-JKAP, JKAP lentivirus; LV-si-JKAP, si-JKAP lentivirus; PBMC, peripheral blood mononuclear cells.
